# Dynamic Urothelial Remodeling in Diabetic Bladder Dysfunction: Disease-Course Changes, Molecular Mechanisms, and Clinical Implications

**DOI:** 10.3390/ijms27209033

**Published:** 2026-10-12

**Authors:** Quan Song, Wanjian Xie, Jing Wang, Yehuan Yuan, Zhi Li, Sisi Sun, Kun Ai

**Affiliations:** School of Acupuncture-Moxibustion, Tuina and Rehabilitation, Hunan University of Chinese Medicine, Changsha 410208, China; 20243685@stu.hnucm.edu.cn (Q.S.); 20243686@stu.hnucm.edu.cn (W.X.); 202530730@stu.hnucm.edu.cn (J.W.); 202530728@stu.hnucm.edu.cn (Y.Y.); 202530729@stu.hnucm.edu.cn (Z.L.); 202630739@stu.hnucm.edu.cn (S.S.)

**Keywords:** diabetic bladder dysfunction, urothelium, urothelial remodeling, NLRP3 inflammasome, ferroptosis, oxidative stress, lower urinary tract dysfunction

## Abstract

Diabetic bladder dysfunction (DBD) includes storage abnormalities and voiding dysfunction, but its clinical and experimental phenotypes do not follow a uniform chronological sequence. The urothelium is a urine barrier and a mechanosensory, paracrine, and inflammatory interface. Early diabetes may cause urothelial shedding, barrier permeability, abnormal ATP release, and altered sensory receptors. With longer disease duration, superficial architecture and some barrier properties may recover, while sensory and paracrine abnormalities persist. In parallel, impaired afferent conduction, reduced detrusor contractility, and extracellular matrix remodeling may become prominent, so local urothelial changes do not necessarily track whole-bladder function. Metabolic and oxidative stress, NLRP3-associated inflammation and pyroptosis, ferroptosis, and epigenetic and post-transcriptional regulation may contribute to urothelial injury, although evidence differs in cell specificity and disease-course localization. This review integrates clinical, animal, and cellular studies to examine urothelial barrier, sensory, paracrine, and cell-fate changes across DBD functional states, appraise the supporting molecular evidence and its limitations, and discuss the clinical relevance of urothelial biomarkers and therapeutic targets.

## 1. Introduction

Diabetes mellitus is a chronic metabolic disease whose global burden continues to rise [1]. According to the 11th edition of the International Diabetes Federation (IDF) Diabetes Atlas, approximately 589 million adults aged 20–79 years were living with diabetes worldwide in 2024, and this number is projected to reach 853 million by 2050 [2]. As survival improves, the long-term consequences of diabetes-related lower urinary tract dysfunction are receiving increasing attention [3]. Diabetic bladder dysfunction (DBD) can affect both storage and voiding and, in severe cases, may lead to recurrent urinary tract infection, urinary retention, or upper urinary tract damage. Although the traditional view has emphasized reduced bladder sensation, increased capacity, and impaired contractility, DBD is now recognized as a clinically heterogeneous condition that cannot be represented solely by a late low-activity phenotype [4].

The “temporal theory” proposed by Daneshgari et al. divides DBD into an early compensated state and a late decompensated state and has provided a useful framework for interpreting disease-course-related changes in bladder function [5]. However, available studies show that storage abnormalities, impaired voiding, and mixed storage–voiding dysfunction do not emerge at consistent time points across patients or animal models [6,7]. DBD therefore cannot be staged on the basis of diabetes duration alone; changes in individual bladder tissues at different functional states must also be considered.

The urothelium provides a tissue-level entry point for examining DBD because it is directly exposed to the diabetic metabolic environment [8]. In addition to forming the barrier between urine and the bladder wall, urothelial cells express transient receptor potential (TRP) channels, P2X/P2Y receptors, and cholinergic receptors and can release adenosine triphosphate (ATP), acetylcholine, prostaglandins, nitric oxide (NO), and neurotrophic factors. Through these mechanisms, the urothelium participates in bladder-filling sensation, local signal transmission, and inflammatory regulation [9,10]. Diabetes-associated abnormalities in urothelial barrier integrity, sensory receptors, and mediator release have been reported separately [11,12], but these changes do not always occur in parallel with alterations in bladder nerves, the detrusor, or the overall urodynamic phenotype. Their patterns across different DBD functional states and their actual contribution to whole-bladder function therefore remain insufficiently integrated. Moreover, molecular studies differ substantially in cellular source and strength of evidence: some findings are directly localized to the urothelium, whereas others are derived from whole-bladder tissue. It is therefore necessary to distinguish mechanisms that can be attributed to urothelial cells from those that cannot and to consider whether their evidence is linked to a specific DBD functional state. Accordingly, this review first summarizes disease-course-related changes in DBD phenotypes and in bladder neural, detrusor, and urethral function; then focuses on urothelial barrier, sensory, paracrine, injury, and repair processes; critically evaluates the evidence supporting metabolic stress, inflammation, cell-death, and epigenetic mechanisms; and finally discusses the potential clinical value and current limitations of the urothelium as a source of biomarkers and therapeutic targets.

## 2. Disease-Course Phenotypes of DBD and Their Tissue-Level Functional Context

### 2.1. Disease-Course Changes in DBD Functional Phenotypes

DBD may present with storage dysfunction, voiding dysfunction, or a combination of both. Clinical manifestations include urgency, urinary frequency, urgency urinary incontinence, reduced urinary flow, prolonged voiding, and incomplete emptying [7]. Urodynamic testing may demonstrate detrusor overactivity (DO), detrusor underactivity (DU), or combined abnormalities of storage and voiding [13]. Thus, DBD extends beyond the traditionally described pattern of reduced bladder sensation and impaired detrusor contractility.

Clinical urodynamic studies in women with DBD, including a systematic review and meta-analysis, have reported increased volume at first bladder sensation, maximum bladder capacity, and post-void residual volume, together with reduced maximum flow rate and detrusor pressure, although findings have not been uniform across studies [14,15,16]. In another study stratified by diabetic microvascular complications, patients without overt complications more often exhibited DO with preserved detrusor contractility; those with retinopathy could show both DO and impaired contractility; and DU was more common among patients with both retinopathy and nephropathy [13]. Comparisons between patients with shorter- and longer-duration type 2 diabetes mellitus (T2DM) similarly showed more voiding difficulty and low-contractility patterns with longer disease duration [17]. More recent work indicates that some patients with long-standing diabetes continue to have reduced voiding efficiency and impaired detrusor contractility despite good current glycemic control [18]. A large population-based analysis of National Health and Nutrition Examination Survey (NHANES) 2007–2018 data also found that associations between diabetes-related metabolic measures and overactive bladder (OAB) were accompanied in part by changes in systemic inflammatory indices, including leukocyte and neutrophil counts, suggesting a potential contribution of systemic metabolic inflammation to diabetes-associated storage symptoms [19]. Because most of these studies are cross-sectional, however, they cannot establish that an individual patient follows a fixed progression from a hyperactive to a low-activity state.

Animal studies further support disease-course-related shifts in DBD phenotype, but the trajectories differ among models. Female Akita mice show increased voiding frequency and bladder hyperactivity at earlier time points, whereas by 30 weeks they exhibit larger voided volumes, increased bladder capacity, and reduced voiding frequency, consistent with a low-activity phenotype [20]. A T2DM rat model induced by a high-fat diet (HFD) combined with low-dose streptozotocin (STZ) shows a similar pattern, with predominantly compensatory functional changes early and progressive deterioration of voiding function as diabetes persists [21]. KK-Ay mice likewise show a hyperresponsive bladder phenotype at 12–18 weeks, followed by reduced voiding efficiency and bladder activity by 25 weeks [22]. These observations support a transition from an early hyperresponsive state to a later low-activity phenotype in some, but not all, diabetic animal models.

Not all models follow this pattern. Goto-Kakizaki rats show relatively limited bladder dysfunction early in the disease and develop increased bladder capacity and reduced voiding pressure only after long-standing diabetes [23]. FVB db/db mice also display marked differences in voiding phenotype at 12, 24, and 52 weeks without a consistent hyperactive-to-low-activity sequence [24,25]. Thus, although disease-course-related changes are evident in DBD, the specific phenotype and the time at which it emerges are strongly model dependent.

These differences may reflect diabetes type, animal strain, sex, age at disease onset, and modeling strategy [6,26,27]. A recent systematic analysis of 352 studies further showed that experimental models of diabetic neurogenic bladder most commonly use STZ alone or STZ combined with high-sugar/high-fat feeding, but vary substantially in animal sex, STZ dose, model duration, and criteria used to define successful modeling [28]. Such heterogeneity helps explain why the timing of “compensated” and “decompensated” states is not consistent across studies. These terms are therefore better used to describe bladder functional status at a given observation point rather than to denote a fixed duration of diabetes or a specific animal age. More broadly, the stage-related heterogeneity of DBD suggests that bladder nerves, the detrusor, the urethra, and the urothelium may each undergo changes of different magnitude at different points in the disease course.

### 2.2. Disease-Course Changes in Neural, Detrusor, and Urethral Function

Changes in DBD phenotype are accompanied by alterations in bladder nerves, detrusor function, and urethral function, but these abnormalities do not necessarily occur synchronously [29]. Neural changes can appear early. Diabetes is associated with alterations in neurotrophic signaling, including nerve growth factor (NGF), at early time points [30], whereas NGF levels in the bladder and L6–S1 dorsal root ganglia may decline with prolonged disease [31,32,33]. Regulation involving proNGF and the p75 neurotrophin receptor (p75NTR), as well as neurotransmitter transport and release, can also become abnormal [34]. At later time points, Goto-Kakizaki rats develop slower bladder afferent conduction and a lower proportion of Aδ fibers, together with increased bladder capacity and reduced voiding pressure [23]. STZ-diabetic mice have also been reported to show reduced neural content in the detrusor and lower microvascular density in the lamina propria [35]. Collectively, these findings suggest that diabetes-associated neural abnormalities may evolve from early changes in neurotrophic and sensory signaling toward later impairment of conduction and bladder innervation [36,37].

Detrusor function can also change over the course of diabetes. In HFD/STZ rats, contractile responses to carbachol and ATP are enhanced during the compensated state; after transition to the decompensated state, ATP-mediated responses remain relatively preserved, whereas the relative cholinergic contractile advantage diminishes [21]. These findings indicate that late detrusor dysfunction does not reflect uniform suppression of all contractile pathways and may instead involve divergent changes in cholinergic and purinergic regulation. Cell-specific genetic studies provide complementary evidence. Smooth muscle-specific deletion of manganese superoxide dismutase (MnSOD) aggravates diabetes-induced oxidative bladder injury and functional abnormalities [38], whereas conditional deletion of the insulin receptor in smooth muscle causes voiding dysfunction, disorganization of the muscle layer, and reduced expression of contractile molecules even without marked systemic hyperglycemia [39]. The former finding indicates that smooth-muscle antioxidant capacity modifies the severity of bladder injury in diabetes, whereas the latter suggests that impaired insulin signaling within detrusor smooth muscle can affect voiding independently of systemic hyperglycemia. Together, these studies support an active contribution of the detrusor to DBD rather than a purely passive response to neural or urothelial injury.

Structural findings from long-term diabetic models and clinical studies provide an additional layer of evidence. Compared with early studies in which altered contractile responsiveness predominates, long-standing diabetes has been associated with increased type I collagen, reduced elastin, and abnormalities in extracellular matrix (ECM)-regulatory molecules, including transforming growth factor-β1 (TGF-β1) and the matrix metalloproteinase-1/tissue inhibitor of metalloproteinases-1 (MMP-1/TIMP-1) system, indicating that later detrusor dysfunction may be accompanied by matrix remodeling [40,41,42,43]. However, most of these studies used whole-bladder tissue rather than cell-specific approaches; the observed ECM changes therefore cannot all be attributed directly to detrusor smooth-muscle cells. Voiding function also depends on more than detrusor contractility alone.

**Figure 1:**
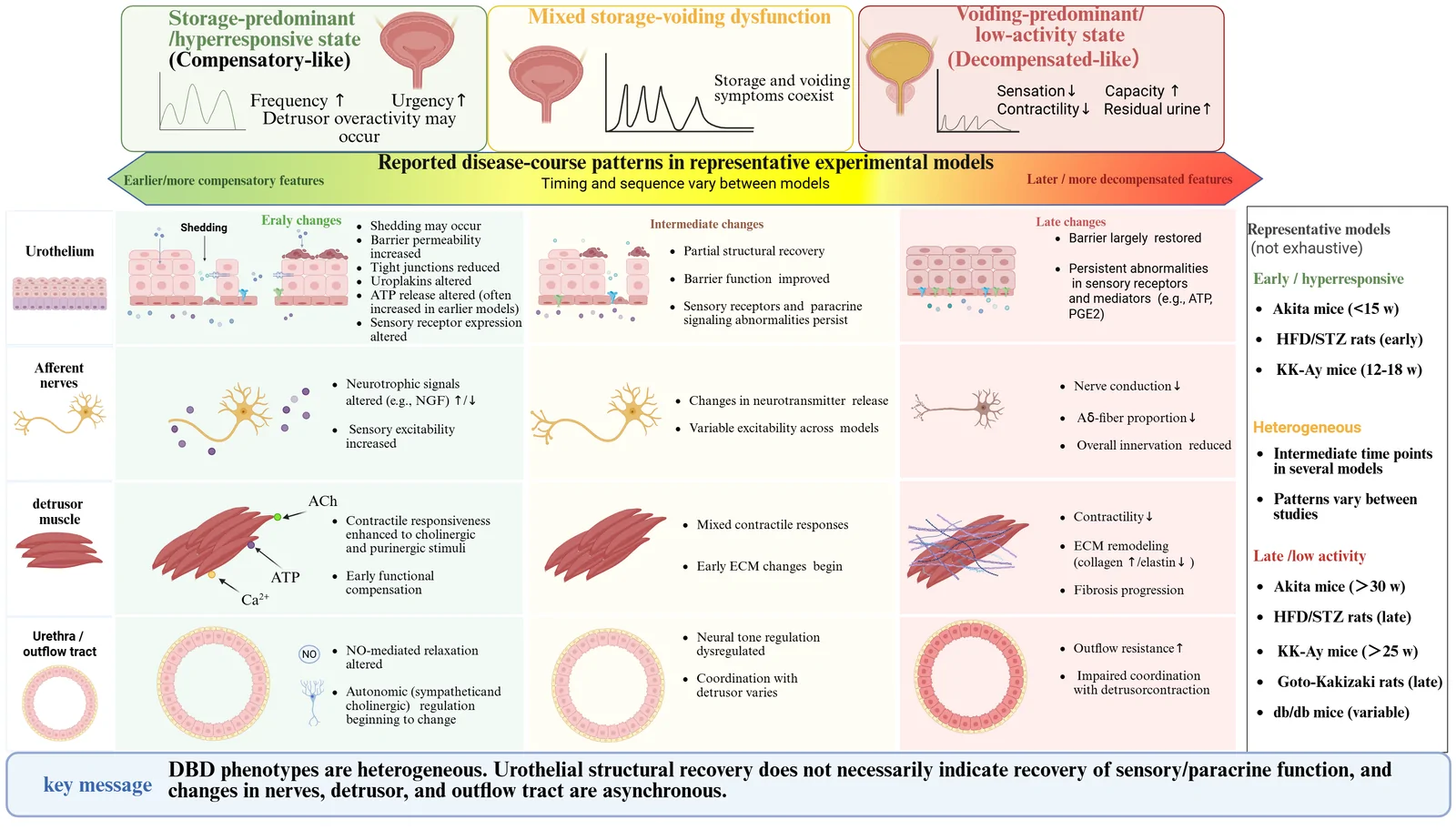
Functional phenotypes and tissue-level changes across the course of diabetic bladder dysfunction. The schematic summarizes storage-predominant/hyperresponsive, mixed or heterogeneous, and voiding-predominant/low-activity functional states together with reported changes in the urothelium, bladder nerves, detrusor, and urethral/outflow tract. These states are functional descriptions rather than fixed chronological stages. In some models, urothelial barrier injury and sensory/paracrine abnormalities are prominent at earlier time points; barrier structure may subsequently improve while selected sensory or mediator-release abnormalities persist. Neural conduction and innervation and detrusor contractility may decline with prolonged diabetes, and urethral regulation may also become abnormal. Representative animal models are shown for context, but trajectories vary according to diabetes type, model, sex, and experimental conditions. Created in BioRender. Song, Q. (2026) https://BioRender.com/r6l6ffs.

Studies of the diabetic urethra have documented changes in NO-dependent relaxation as well as sympathetic and cholinergic neural regulation [44]. Consequently, impaired bladder emptying in long-standing DBD may reflect both insufficient detrusor contraction and abnormal regulation of the urethral outlet; reduced detrusor contractility alone cannot explain the full spectrum of voiding dysfunction [45]. Overall, disease-course-related changes in DBD are accompanied by alterations in bladder nerves, the detrusor, and the urethra that differ in both timing and severity [46]. The major DBD functional states and corresponding changes in the urothelium, bladder nerves, detrusor, and urethra are summarized in Figure 1.

## 3. Tissue and Cellular Remodeling of the Bladder Urothelium in Diabetes

The urothelium is directly exposed to urinary glucose, osmotic changes, and diabetes-related metabolites while also performing barrier, bladder-filling sensory, and local mediator-release functions [47,48]. It can therefore be directly affected by the diabetic environment, and its structural and functional changes may in turn influence adjacent bladder nerves and the detrusor [49,50]. Importantly, studies conducted at different disease time points indicate that urothelial injury does not simply worsen continuously during DBD. Barrier disruption and sensory and paracrine abnormalities can occur early; with longer disease duration, superficial architecture and barrier function may partially recover, whereas some sensory and mediator-release abnormalities persist [11,51]. This disease-course pattern makes the urothelium an important tissue context for understanding functional heterogeneity in DBD. The following sections address barrier structure, sensory and paracrine function, and cellular injury and repair.

### 3.1. Urothelial Barrier Structure and Permeability

The normal urothelial barrier depends largely on apical uroplakin plaques in umbrella cells, tight junctions, and adherens junctions, which restrict the passage of urinary constituents into the bladder wall [52]. Early diabetes can be associated with urothelial cell shedding, disruption of intercellular junctions, and increased barrier permeability [11,53]. In Akita mice at 15 weeks, during an OAB phenotype, focal urothelial shedding, increased permeability, and reduced barrier-associated proteins are evident; deletion of NOD-like receptor family pyrin domain-containing 3 (NLRP3) markedly attenuates these changes. By 30 weeks, when the bladder displays a low-activity phenotype, urothelial permeability and some barrier-associated proteins have returned toward control levels despite persistent whole-bladder dysfunction. This mismatch indicates that restoration of the urothelial barrier does not equate to recovery of bladder function and that the late DBD phenotype cannot be explained solely by the severity of urothelial barrier injury [51]. Human bladder biopsy studies provide complementary evidence: patients with diabetes and OAB show reduced E-cadherin expression and increased suburothelial activated mast cells, although several barrier and inflammatory markers do not differ significantly from those in patients with non-diabetic OAB [54]. These findings suggest that some abnormalities may be shared features of OAB rather than diabetes-specific urothelial injury.

In addition to high-glucose-associated injury, insulin receptor signaling may contribute to normal maintenance of the urothelial barrier. A 2024 study showed that selective deletion of the insulin receptor in superficial and intermediate urothelial cells reduced expression of the barrier-associated molecules CDH1 and TJP1 and increased urothelial permeability [55]. Because that study primarily assessed susceptibility to urinary tract infection and did not evaluate a DBD functional phenotype, the findings support a role for insulin receptor signaling in urothelial barrier maintenance but do not yet establish its specific contribution to barrier remodeling in DBD.

### 3.2. Abnormal Urothelial Sensory Transduction and Paracrine Function

The urothelium senses bladder filling and mechanical stretch through purinergic, cholinergic, and TRP-related receptors and releases ATP, acetylcholine, prostaglandins, and NO, thereby modulating bladder nerves and detrusor function [56,57,58]. Diabetes can alter this process, but the direction and timing of changes differ among mediators [59]. In an STZ rat study, stretch-induced ATP release was markedly increased at 2 weeks of diabetes but was no longer significantly different from controls at 12 weeks; acetylcholine release did not show the same pattern [49]. Another T2DM study found increased urothelial ATP release and vesicular nucleotide transporter (VNUT) expression together with reduced bladder blood flow, enhanced oxidative stress, and inflammation; interventions that improved these abnormalities also attenuated the ATP-related changes [60]. These findings indicate that abnormalities in ATP release are both disease-course- and mediator-specific and may be shaped by the local metabolic and tissue environment.

Urothelial sensory receptors can also remain abnormal across disease time points. STZ rats show altered expression of transient receptor potential vanilloid 1 (TRPV1), P2X2, P2X3, and muscarinic M2 and M3 receptors at different stages of diabetes [11]. A T2DM model with OAB further demonstrated increased TRPV1 and P2X3 in the urothelial region, accompanied by elevated urinary ATP and storage dysfunction [61]. Human bladder biopsies similarly showed changes in sensory-associated proteins, including M3 and P2X3, in patients with diabetes and OAB; however, some markers were comparable to those in non-diabetic OAB [54]. Sensory receptor abnormalities may therefore contribute to diabetes-associated storage symptoms, but some changes may reflect OAB more generally rather than diabetes-specific pathology. In addition, molecular measurements in several animal studies were obtained from whole-bladder tissue, and their precise cellular origin remains to be established.

Prostaglandin studies further demonstrate that urothelial paracrine abnormalities do not necessarily diminish in parallel with declining whole-bladder function. At 30 weeks, female Akita mice already display a low-activity bladder phenotype, yet stretch-induced urothelial prostaglandin E2 (PGE2) release remains increased while detrusor PGE2 release is reduced. Prostaglandin F2α (PGF2α) release is not significantly increased, but detrusor FP receptor expression and FP receptor-mediated contractile responses are enhanced, and these changes are NLRP3 dependent [62]. Thus, late DBD is not characterized by a uniform reduction in urothelial sensory and paracrine signaling; mediator release, sensory receptors, and detrusor responses can change in different directions. Together with the barrier findings described above, these observations show that structural recovery of the urothelium does not necessarily imply recovery of its sensory or paracrine functions.

### 3.3. Urothelial Cell Death, Phenotypic Transition, and Repair

Diabetes also affects urothelial cell survival and renewal. Animal studies have documented umbrella-cell shedding, focal cell loss, and ultrastructural abnormalities [63]. With longer disease duration, superficial cells can repopulate the luminal surface and restore basic urothelial stratification [11,63]. The diabetic urothelium therefore does not undergo continuous cell loss; injury occurs in parallel with replenishment and repair, and restoration of the superficial cell layer does not establish that the newly formed urothelium has fully recovered normal function.

The most direct evidence for regulated cell death in the diabetic urothelium currently concerns pyroptosis and ferroptosis. Diabetic animals and high-glucose-treated urothelial cells show NLRP3-associated cell injury and pyroptotic changes [51,64]. More recent studies have also identified ferroptosis-related abnormalities in human DBD tissue, diabetic animals, and high-glucose-treated cells [65]. By contrast, much of the evidence for apoptosis derives from whole-bladder tissue, and urothelial cell-specific evidence remains limited [66]. Multiple forms of cell death may therefore contribute to urothelial injury in diabetes, although their specific molecular regulation requires separate evaluation.

Beyond cell death, diabetes can alter urothelial cell phenotype. Human diabetic bladder tissue, diabetic animal models, and high-glucose-treated cells show weakened epithelial junctions, reduced epithelial markers, and increased mesenchymal-associated markers, consistent with an epithelial-to-mesenchymal transition (EMT)-like phenotypic shift [67]. Increased senescence-associated β-galactosidase (SA-β-gal) has also been observed in the diabetic urothelium, although whether cellular senescence directly impairs urothelial repair or contributes to DBD progression remains unclear [68].

**Figure 2:**
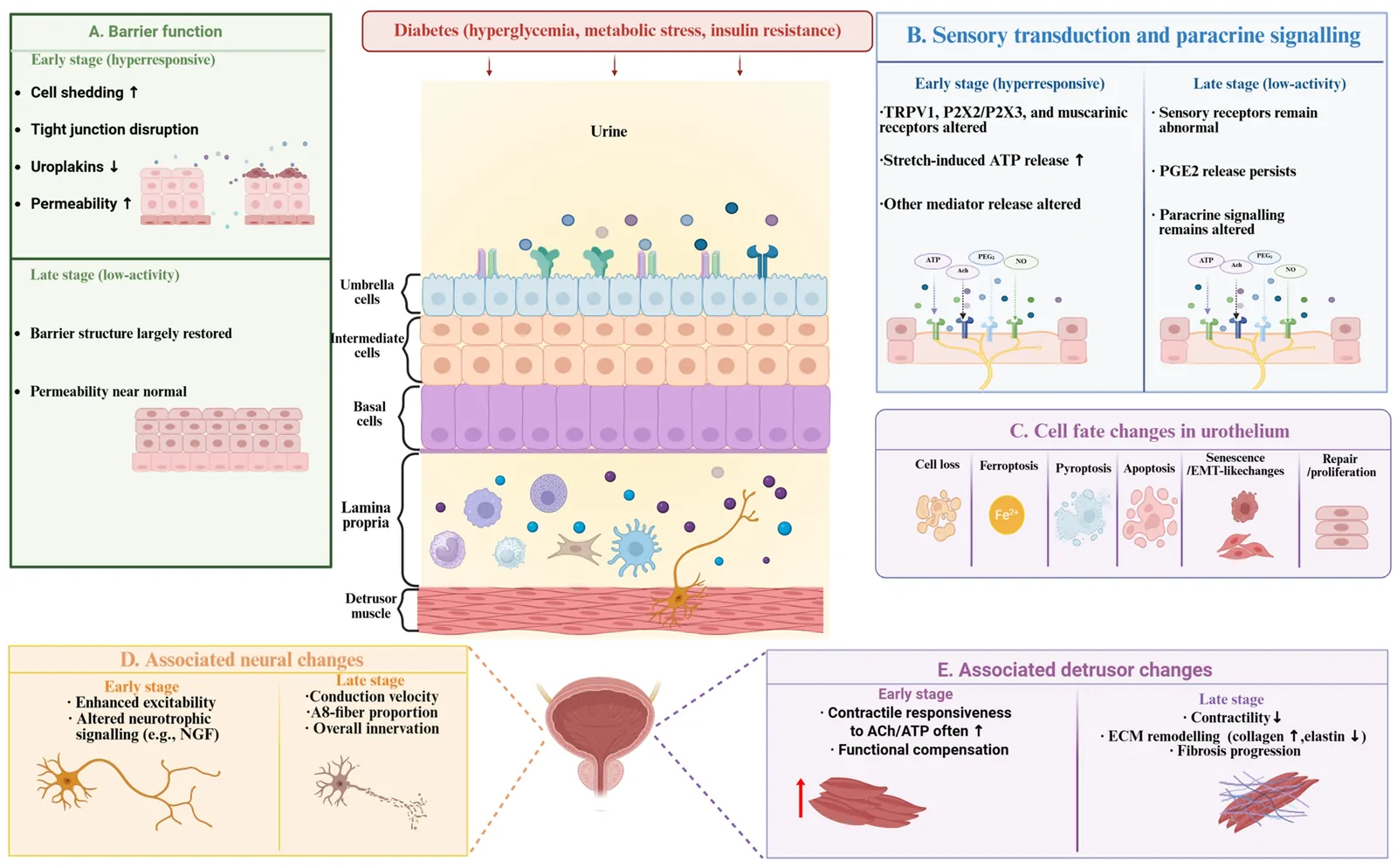
Structural and functional remodeling of the urothelium in diabetic bladder dysfunction and associated neural and detrusor changes. The central schematic depicts the urothelial layers, lamina propria, bladder nerves, and detrusor. (**A**) Early barrier abnormalities include urothelial shedding, junctional disruption, reduced uroplakin-associated barrier integrity, and increased permeability, whereas superficial structure and permeability may partly recover at later time points. (**B**) Sensory and paracrine abnormalities include changes in TRPV1, P2X2/P2X3, muscarinic receptors, ATP release, and prostaglandin signaling; these changes are mediator- and disease-course-specific. (**C**) Reported urothelial cell-fate changes include cell loss, pyroptosis, ferroptosis, apoptosis, senescence/EMT-like changes, and repair or proliferation, although the strength of urothelial-specific evidence differs among mechanisms. (**D**) Diabetes-associated neural changes include altered neurotrophic signaling and, later, reduced afferent conduction and innervation. (**E**) Detrusor responses may be enhanced in some compensated models and decline with prolonged diabetes, accompanied by extracellular matrix remodeling. Structural recovery of the urothelial barrier does not necessarily indicate normalization of sensory or paracrine function. Created in BioRender. Song, Q. (2026) https://BioRender.com/vs5dkuq.

Overall, the diabetic urothelium exhibits barrier injury and repair, abnormalities in sensory and paracrine function, and changes in cell injury and renewal across different disease time points, but these processes do not show a uniform temporal relationship with whole-bladder function, bladder nerves, or detrusor changes. Table 1 summarizes disease-course characteristics across commonly used diabetic animal models and compares the correspondence among bladder phenotype, urothelial changes, and neuromuscular abnormalities. Figure 2 provides an integrated overview of urothelial barrier, sensory, paracrine, and cell-fate changes and their relationship to bladder nerves and the detrusor.

## 4. Molecular Mechanisms of Pathological Urothelial Remodeling in Diabetes

### 4.1. Metabolic Stress, Oxidative Stress, and Mitochondrial Injury

Persistent hyperglycemia can alter urothelial energy metabolism and redox homeostasis. At different disease time points, STZ rats show abnormal urothelial expression of glucose transporter 1 (GLUT1) and enzymes associated with the polyol pathway [11]. Tissue-layer-specific analyses further indicate that diabetes does not affect the urothelium and detrusor in the same metabolic manner: urothelial changes primarily involve glycolysis, lipid metabolism, and prostaglandin metabolism, whereas detrusor changes more strongly involve the polyol pathway, pentose phosphate pathway, tricarboxylic acid cycle, and fatty-acid oxidation [70]. Proteomic studies likewise identify suppression of the nuclear factor erythroid 2-related factor 2 (NRF2)-associated antioxidant response as a prominent feature of the diabetic urothelium, whereas detrusor changes are more concentrated in cytoskeletal and contractile proteins [71]. These findings indicate that diabetes produces tissue-specific metabolic effects within the bladder and that the urothelium has a distinct metabolic and antioxidant response profile.

Consistent with these metabolic changes, oxidative and nitrosative stress is increased in the diabetic urothelium. In STZ-diabetic mice, urothelial 3-nitrotyrosine and nitric oxide synthase (NOS)-related signaling are increased and accompanied by activation of extracellular signal-regulated kinase (ERK) and p38, whereas some other stress responses are more prominent in the detrusor [72]. Together with the reduced NRF2-associated antioxidant response, these findings indicate a shift in the diabetic urothelium away from normal redox balance toward oxidative stress [71]. Other DBD studies have reported abnormalities in protein degradation pathways [29,73] and increased reactive oxygen species (ROS) generation involving the NADPH oxidase (NOX) family [74]. In animal models, interventions targeting NOX4/RAC1, NRF2, and the methylglyoxal–advanced glycation end product–receptor for advanced glycation end products (MGO–AGE–RAGE) axis can attenuate diabetic bladder oxidative injury and improve bladder function [75,76,77]. However, these intervention studies are not uniformly urothelial-specific, because several molecular readouts were obtained from whole-bladder tissue or the detrusor. They support a role for oxidative stress in DBD but do not, by themselves, establish that urothelial pathway modulation mediates the functional benefit. Overall, oxidative stress is implicated in diabetic bladder injury, while the causal contribution of individual urothelial oxidative-stress pathways to inflammation, cell death, and bladder dysfunction requires further cell-specific functional validation.

Long-standing diabetes can also affect urothelial mitochondria. In long-term STZ-diabetic rats, urothelial mitochondria show vacuolar degeneration together with endoplasmic reticulum swelling, reduced cell junctions, and cellular degeneration; similar mitochondrial ultrastructural abnormalities can also be observed in other layers of the bladder wall [63]. Current evidence for urothelial mitochondrial abnormalities is therefore based mainly on ultrastructural and oxidative-stress findings. Direct functional assessments of mitochondrial membrane potential, oxygen consumption, respiratory-chain activity, and ATP production remain scarce, and the specific contribution of mitochondrial dysfunction at different DBD functional states has not been established.

### 4.2. Inflammasome Activation and Pyroptosis

The NLRP3 inflammasome is a cytosolic immune complex composed of NLRP3, apoptosis-associated speck-like protein containing a CARD (ASC), caspase-1, and associated components. Its activation promotes maturation of interleukin-1β (IL-1β) and IL-18 and induces gasdermin D (GSDMD)-mediated inflammatory pyroptosis [78,79]. Among inflammatory mechanisms studied in DBD, NLRP3 currently has comparatively strong urothelial evidence. Diabetes-related metabolites can activate NLRP3 in primary urothelial cells, and urothelial NLRP3 activation is observed early in Akita mice [69]. NLRP3 deficiency attenuates bladder inflammation and some urodynamic abnormalities while also reducing urothelial barrier injury and changes in the proportions of Aδ and C fibers [51,69]. However, the whole-animal deletion used in these studies does not isolate the urothelial contribution to the functional benefit. In Akita mice, marked urothelial permeability and cell shedding are present during the OAB state, whereas some barrier abnormalities are attenuated after the bladder develops a low-activity phenotype [20,51]. Taken together, current evidence supports an important contribution of NLRP3-associated inflammation to early urothelial injury and sensory-related changes in DBD [80], whereas the late low-activity phenotype also reflects persistent injury in neural and detrusor compartments.

High-glucose-induced oxidative stress can further promote NLRP3 inflammasome activation and pyroptosis-related processes [81]. Increased ROS facilitates the interaction of thioredoxin-interacting protein (TXNIP) with NLRP3, followed by caspase-1 activation, GSDMD cleavage, and increased maturation and release of IL-1β and IL-18 [82]. In a metformin intervention study, attenuation of TXNIP-, NLRP3-, and GSDMD-related abnormalities was accompanied by improvement in urothelial inflammation and barrier injury, providing relatively direct evidence that the TXNIP–NLRP3–caspase-1–GSDMD pyroptotic axis contributes to diabetic urothelial injury [64]. Beyond the inflammasome, high glucose can promote nuclear translocation of nuclear factor kappa B (NF-κB) p65 through allograft inflammatory factor-1 (AIF-1), with concomitant increases in ROS, interleukin-6 (IL-6), and tumor necrosis factor-α (TNF-α); AIF-1 knockdown attenuates these responses, supporting a contribution of NF-κB-associated inflammation to high-glucose-induced urothelial injury [83]. Importantly, NF-κB has context-dependent functions in the urothelium. Sustained overactivation under high-glucose conditions can promote inflammation, whereas a basal level of NF-κB signaling participates in the normal expression of barrier proteins and antimicrobial peptides [55]. NF-κB activation should therefore not be interpreted uniformly as a marker of urothelial injury.

### 4.3. Ferroptosis and Other Cell-Fate Regulatory Mechanisms

Ferroptosis is a regulated form of cell death characterized by iron-dependent accumulation of lipid peroxidation, in which reduced NRF2/glutathione peroxidase 4 (GPX4)-mediated antioxidant defense is a central event [84,85,86]. Recent work in human bladder tissue from patients with DBD, diabetic animals, and high-glucose-treated bladder epithelial cells has shown reduced NRF2 and GPX4 together with increased intracellular iron, lipid ROS, and malondialdehyde (MDA); inhibition of ferroptosis attenuates cell injury and inflammation [65]. Compared with other cell-death pathways, ferroptosis currently has urothelial-related evidence at the human tissue, animal, and cellular levels. Earlier urothelial proteomic work also identified suppression of NRF2-associated antioxidant responses in diabetes, which is directionally consistent with these findings [71]. However, that study did not directly assess intracellular iron, lipid peroxidation, or other ferroptosis markers and should therefore be viewed as complementary evidence for NRF2 dysregulation rather than direct evidence of ferroptosis. The upstream regulation of ferroptosis by H3K9 lactylation, FTO, and N6-methyladenosine (m6A) is discussed below.

Apoptosis may also contribute to diabetic bladder injury. Classical apoptosis includes intrinsic and extrinsic pathways; the mitochondrial pathway is regulated largely by the BCL-2 family and culminates in caspase activation [87,88]. Direct evidence in DBD urothelial cells remains limited. In whole-bladder tissue from STZ-diabetic rats, the Bcl-2/Bax ratio is reduced, caspase-3 activity is increased, and apoptosis-inducing factor (AIF) translocates from mitochondria to the nucleus; inhibition of poly(ADP-ribose) polymerase (PARP) attenuates these changes and improves bladder function [66]. Because these observations were derived mainly from whole-bladder tissue, they do not establish that PARP-associated mitochondrial apoptosis occurs predominantly in the urothelium. Persistent elevation of BNIP3 together with cell degeneration and death has been observed in the diabetic urothelium [11], but these findings alone are insufficient to define a specific apoptotic pathway.

Evidence for autophagy and mitophagy in the DBD urothelium is even more limited. Autophagy is a dynamic lysosomal degradation process, and changes in microtubule-associated protein 1 light chain 3 (LC3) or autophagosome abundance alone cannot distinguish increased autophagosome formation from impaired degradation; such measures are therefore insufficient to determine autophagic flux [89]. Autophagosomes have been observed in the urothelium of animals with long-standing diabetes, but their number alone cannot establish abnormal autophagic flux or confirm mitophagy. Mitophagy is mediated principally by ubiquitin-dependent PTEN-induced kinase 1 (PINK1)/Parkin signaling and receptor-mediated pathways involving FUN14 domain-containing 1 (FUNDC1), BNIP3/NIX, and related proteins [90,91]. These mechanisms have received increasing attention in other diabetic complications [91], and PINK1/Parkin-associated mitophagy has been studied in the DBD detrusor and bladder smooth-muscle cells [92]. However, direct validation in the DBD urothelium using cell-specific localization, flux measurements, and functional intervention is lacking. At present, ferroptosis therefore has comparatively direct experimental support in the urothelium, whereas apoptosis, autophagy, and mitophagy require more definitive cell-specific and functional evidence.

### 4.4. Epigenetic and Post-Transcriptional Regulation

Current studies of epigenetic and RNA regulation in the DBD urothelium have focused mainly on histone lactylation and m6A modification. Lactylation links changes in lactate metabolism to gene regulation, whereas m6A affects RNA stability and translation through dynamic interactions among writer, eraser, and reader proteins; both processes have been implicated in diabetes-associated inflammation, oxidative stress, and tissue injury [93,94,95]. High glucose increases intracellular lactate and histone H3 lysine 9 lactylation (H3K9la) and promotes expression of the fat mass and obesity-associated protein (FTO). Increased FTO reduces m6A modification of NRF2 transcripts and weakens the stabilizing effect of insulin-like growth factor 2 mRNA-binding protein 2 (IGF2BP2) on NRF2 mRNA, ultimately reducing NRF2 and antioxidant defense. FTO inhibition restores NRF2 expression and attenuates ferroptosis and inflammation, whereas subsequent NRF2 inhibition reverses this protection [65]. Thus, high-glucose-induced metabolic disturbance can impair urothelial antioxidant capacity through an H3K9la–FTO–m6A–NRF2 axis, providing an upstream regulatory mechanism for the ferroptotic process described above.

This mechanism is broadly consistent with the emerging lactylation literature in other diabetic complications. Recent reviews indicate that lactate accumulation and protein lactylation can contribute to diabetes-associated inflammation, oxidative stress, and fibrosis, although their effects vary by tissue and molecular target [93,96]. The H3K9la–FTO–m6A–NRF2 axis identified in DBD therefore provides evidence that metabolic reprogramming can influence urothelial injury through epigenetic and post-transcriptional mechanisms, but whether this pathway varies across DBD functional states remains unknown. Non-coding RNAs may also contribute to diabetic bladder remodeling. MicroRNAs (miRNAs) and exosome-mediated intercellular signaling have been implicated in inflammatory and ECM regulation in diabetic complications [97]. In long-standing diabetic bladder fibrosis, circulating exosomal miR-16-5p and let-7e-5p are reduced and are associated with fibroblast differentiation and changes in ECM-related genes [98]. However, that study focused on circulating exosomes and bladder fibroblasts; it does not establish that these miRNAs originate from the urothelium or directly regulate urothelial pathology.

**Figure 3:**
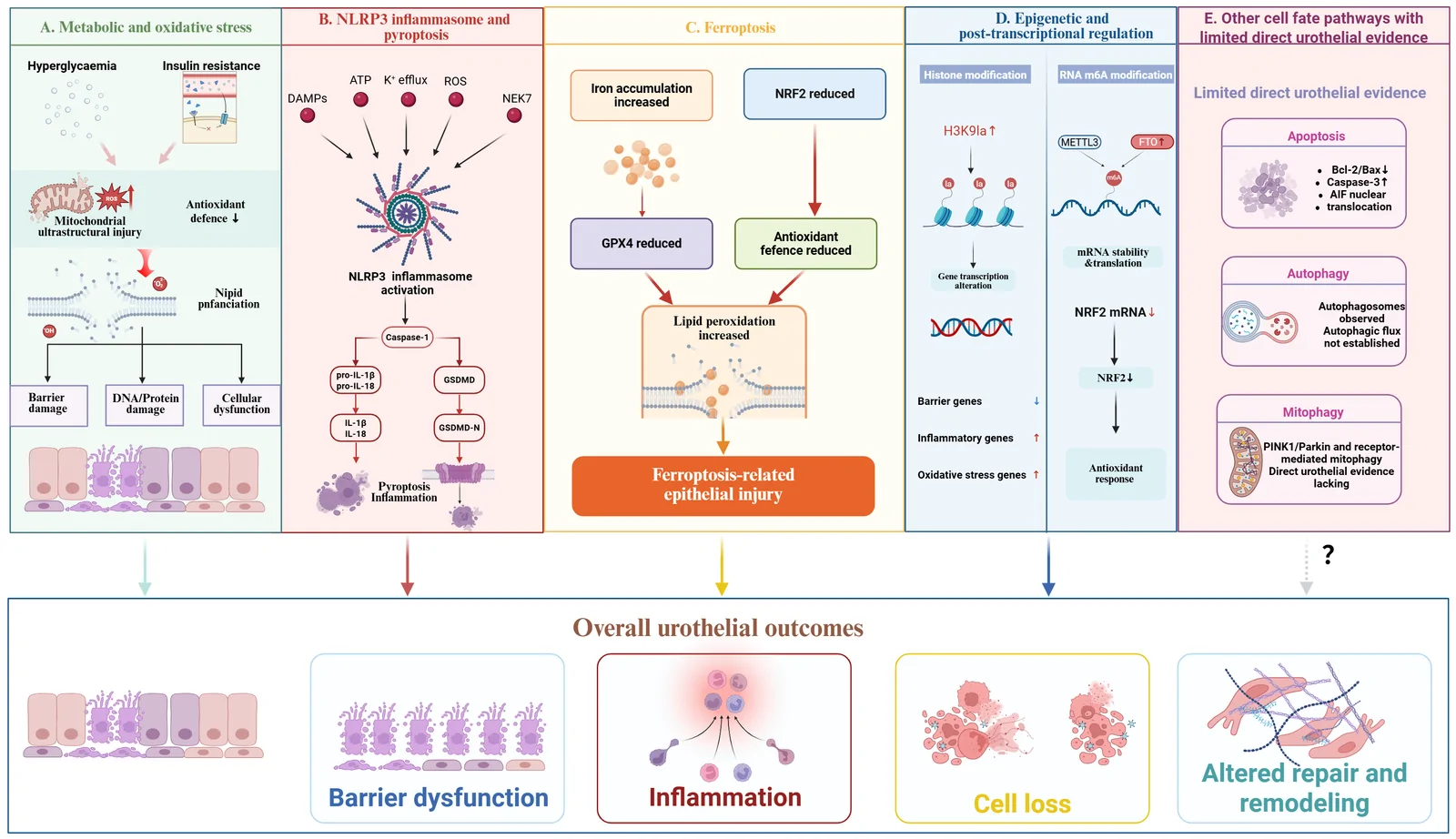
Molecular mechanisms implicated in pathological urothelial remodeling in diabetic bladder dysfunction. (**A**) Hyperglycemia-associated metabolic disturbance is linked to oxidative and nitrosative stress, impaired antioxidant defense, lipid peroxidation, and mitochondrial ultrastructural injury; NOX-related ROS generation and MGO–AGE–RAGE signaling have been implicated in diabetic bladder oxidative injury, although some evidence derives from whole-bladder or detrusor tissue. (**B**) ROS-dependent TXNIP–NLRP3–caspase-1–GSDMD signaling provides comparatively direct evidence for urothelial pyroptosis and inflammatory injury; (**C**) Ferroptosis is characterized by reduced NRF2/GPX4 defense, iron accumulation, and lipid peroxidation and is supported by human tissue, animal, and cell-based evidence. (**D**) High-glucose-induced H3K9 lactylation can increase FTO, reduce m6A modification and IGF2BP2-mediated stabilization of NRF2 mRNA, and thereby suppress NRF2-dependent antioxidant defense, providing an upstream regulatory mechanism for ferroptosis. (**E**) Apoptosis, autophagy, and mitophagy are included as candidate cell-fate pathways for which direct urothelial evidence in DBD remains limited. Canonical pathway components shown for orientation should not be interpreted as uniformly validated in DBD urothelium.The question mark denotes uncertainty regarding the direct contribution of these candidate pathways to overall urothelial outcomes, reflecting the limited direct urothelial evidence in DBD. Created in BioRender. Song, Q. (2026) https://BioRender.com/3910fbk.

Taken together, pathological urothelial remodeling in diabetes involves metabolic and oxidative stress, inflammatory responses, regulated cell death, and epigenetic and post-transcriptional regulation, but the direct urothelial evidence and the strength of association with DBD functional changes differ substantially among mechanisms. Figure 3 summarizes the principal molecular mechanisms and their relative levels of evidence, whereas Table 2 further compares the experimental basis, disease-course relevance, and current limitations of each mechanism.

## 5. Clinical Implications and Therapeutic Potential

Because the urothelium directly contacts urine and contributes to barrier maintenance, bladder sensory transduction, and local mediator release, its pathological changes have potential value for clinical phenotyping and therapeutic development. Current clinical assessment of DBD still relies primarily on lower urinary tract symptoms, uroflowmetry and urodynamics, post-void residual volume, diabetes duration, glycated hemoglobin A1c (HbA1c), and diabetic peripheral neuropathy [13,14,15,16,17,18]. These measures can identify bladder dysfunction and related risk factors but largely reflect whole-bladder function or systemic metabolic and neural injury rather than the status of urothelial barrier, sensory, or paracrine function. Because the urothelium directly faces the bladder lumen, urine is a comparatively accessible biospecimen. Abnormal urothelial ATP release has been observed in diabetic animals [49,60], and prostaglandins participate in urothelial regulation of bladder sensation and contractility [56,62]. Long-standing diabetic bladder fibrosis has also been associated with circulating exosomal miR-16-5p and let-7e-5p [98]. These findings provide a rationale for evaluating urinary proteins, ATP, lipid mediators, oxidative-stress products, and extracellular vesicles as candidate biomarkers. However, no clinically validated marker currently specifically reflects urothelial pathology while distinguishing among DBD functional phenotypes. In addition, inflammatory mediators, metabolites, and extracellular vesicles in urine or the circulation can originate from multiple cell types and tissues, and their urothelial specificity should therefore be interpreted cautiously.

Urothelial molecular abnormalities suggest potential therapeutic targets, but evidence for urothelial-specific therapeutic action varies among pathways and interventions. Metformin reduces TXNIP-, NLRP3-, and GSDMD-related changes and is accompanied by improvement in urothelial inflammation and barrier injury, providing experimental support for NLRP3-associated inflammation and pyroptosis as intervention targets [64]. A phosphodiesterase type 5 (PDE5) inhibitor improves storage and voiding function in T2DM rats together with bladder blood flow, oxidative stress, and ATP-release abnormalities [60]. In addition, interventions directed at NOX-related ROS production, NRF2-dependent antioxidant defense, and MGO–AGE–RAGE signaling can attenuate diabetic bladder oxidative injury and improve selected functional outcomes [74,75,76,77]. However, some of these studies measured molecular effects in whole-bladder tissue or the detrusor, and the therapeutic benefit cannot be assumed to arise primarily from urothelial targeting.

Recent work on regulated cell death has expanded the range of candidate urothelial targets. Reduced NRF2/GPX4, iron accumulation, and increased lipid peroxidation have been observed in human DBD bladder tissue, diabetic animals, and high-glucose-treated bladder epithelial cells; inhibition of ferroptosis reduces both cellular injury and inflammation [65]. The same study showed that increased H3K9 lactylation under high-glucose conditions promotes FTO expression and decreases m6A modification of NRF2 transcripts, thereby reducing NRF2 stability; FTO inhibition or restoration of NRF2 attenuates ferroptosis and inflammation [65]. Compared with apoptosis and mitophagy, for which urothelial-specific evidence remains limited, NLRP3-associated inflammation/pyroptosis and NRF2-associated ferroptosis therefore have more direct experimental support in the urothelium. Nevertheless, these data are still derived predominantly from animal and cellular studies and do not establish therapeutic efficacy in patients with DBD.

A further limitation of current intervention studies is uncertainty regarding the cellular site of drug action. DBD involves the urothelium, bladder nerves, microvasculature, and detrusor; improvement in urodynamic outcomes after a drug intervention does not demonstrate that the urothelium is the principal site of action. For example, several antioxidant interventions improve overall bladder function, but the corresponding molecular readouts were obtained from whole-bladder tissue or the detrusor [74,75,76,77]. Accordingly, pathways supported primarily by these readouts remain candidate mechanisms of DBD and require additional evidence before they can be considered validated urothelial therapeutic targets. Future validation should combine direct urothelial molecular or cellular measurements with urothelial-specific perturbation and assessment of afferent signaling and urodynamic function to test whether the urothelial effect mediates functional improvement.

Differences among DBD functional states should also be considered when evaluating therapy. In some models, urothelial barrier injury at earlier time points diminishes or recovers with longer disease duration [11,51], whereas abnormalities in sensory receptors and paracrine mediators such as PGE2 can persist [11,62]. In parallel, long-standing diabetes can be accompanied by slower afferent conduction, fewer Aδ fibers, reduced detrusor contractility, and ECM remodeling [21,23,40]. The relative contribution of urothelial pathology may therefore differ across functional states, and correction of urothelial inflammation or barrier injury alone may be insufficient to restore late voiding function. Clinical evidence for stratifying urothelium-directed treatment by DBD functional phenotype is currently lacking, and individual molecular targets should not be mapped directly onto fixed durations of diabetes.

Interventions targeting urothelial inflammation must also account for barrier integrity and antimicrobial defense. Patients with diabetes are at increased risk of urinary tract infection [3], and deletion of the urothelial insulin receptor reduces NF-κB-related signaling together with barrier-protein and antimicrobial-peptide expression, thereby increasing susceptibility to infection [55]. Conversely, diabetes and high-glucose conditions can increase NLRP3- and NF-κB-associated inflammatory signaling in association with urothelial barrier injury and inflammation [51,69,83]. Therapeutic modulation of NLRP3 or NF-κB should therefore not be evaluated solely by the magnitude of inflammatory suppression; effects on barrier integrity and local antimicrobial defense must also be assessed.

Overall, current studies provide an experimental basis for urothelial biomarkers and therapeutic targets, but translational evidence remains dominated by animal and cellular work. ATP, prostaglandins, and extracellular vesicles warrant further evaluation as candidate biomarkers [49,56,62,98], whereas NLRP3-associated inflammation/pyroptosis, oxidative stress, and NRF2-associated ferroptosis have experimental intervention data [60,64,65,74,75,76,77]. No clinical biomarker currently specifically reports the urothelial state in DBD, and no clinical intervention has yet targeted a defined urothelial molecular pathway. At present, the urothelium is therefore best viewed as a candidate tissue for DBD phenotyping and therapeutic research rather than as a treatment target independent of bladder nerves and the detrusor.

## 6. Discussion

Available evidence indicates that the urothelium in DBD does not simply undergo progressively worsening structural injury. Rather, barrier function, sensory transduction, paracrine signaling, cell injury, and repair show distinct disease-course-related patterns. Importantly, these changes do not consistently parallel whole-bladder function. In some diabetic models, early urothelial abnormalities include increased permeability, altered ATP release, changes in sensory-associated receptors, and local inflammation and coincide temporally with a hyperresponsive bladder phenotype. With longer disease duration, superficial architecture and some barrier properties may recover while sensory-receptor and paracrine abnormalities persist, even as whole-bladder function shifts toward a low-activity state [11,20,49,51,62]. Current evidence therefore supports the urothelium as an important participant in DBD pathophysiology but does not justify explaining the overall bladder phenotype at different stages solely by the severity of urothelial injury.

The role of the urothelium in early storage dysfunction is supported by a comparatively larger experimental evidence base. Early diabetic models show urothelial barrier injury, increased stretch-induced ATP release, and abnormalities in sensory-associated receptors such as TRPV1, P2X2, and P2X3, which are temporally associated with a hyperresponsive phenotype in some models [11,20,49,51,61]. NLRP3 deficiency can simultaneously attenuate urothelial barrier injury, bladder inflammation, nerve-fiber-related changes, and selected urodynamic abnormalities [51,69]. These findings suggest that urothelial inflammation, barrier dysfunction, and sensory abnormalities may contribute to early storage-phase dysfunction. However, most studies demonstrate parallel changes rather than a complete causal chain, and studies combining urothelial-specific intervention with simultaneous recording of afferent activity and urodynamic function remain lacking. It therefore cannot yet be concluded that urothelial abnormalities are the direct upstream cause of early detrusor overactivity, nor can concurrent contributions from polyuria, systemic metabolic abnormalities, microvascular changes, and neural dysfunction be excluded.

Findings at later disease time points further show that the relationship between urothelial changes and whole-bladder function is not linear. Both STZ and Akita models indicate that superficial urothelial structure or barrier function injured earlier in the disease can partially recover later, whereas abnormalities in selected sensory receptors and paracrine mediators, including PGE2, may persist [11,51,62]. Structural recovery therefore does not establish recovery of urothelial sensory or mediator-release function. Moreover, when the bladder already displays increased capacity, reduced sensation, or a low-activity phenotype, some urothelial signals may remain abnormal or even enhanced [20,62]. Local urothelial signaling abnormalities should therefore not be equated directly with whole-bladder hyperresponsiveness. With persistent diabetes, slower bladder afferent conduction, a lower proportion of Aδ fibers, and reduced detrusor contractility can emerge [23], suggesting that later DBD phenotypes may increasingly reflect neural and detrusor injury. Current evidence thus supports differing contributions of the urothelium, sensory nerves, and detrusor across disease states but is insufficient to establish a causal sequence in which urothelial abnormalities directly drive the transition from a hyperactive to a low-activity bladder.

These findings also argue for a cautious interpretation of the traditional DBD “temporal theory.” Akita, HFD/STZ, and KK-Ay models support a shift from early hyperresponsiveness toward later low activity [20,21,22], whereas Goto-Kakizaki and FVB db/db models do not show the same fixed trajectory [23,24,25]. Studies differ substantially in diabetes type, animal strain, sex, age, STZ dose, and observation time [6,26,27,28]. “Compensated” and “decompensated” are therefore more appropriately used as descriptions of bladder functional state at a specific observation point than as labels for fixed durations of diabetes. Likewise, urothelial barrier injury, repair, and sensory/paracrine abnormalities should not be interpreted as a universal temporal sequence across all DBD models. Future experimental and clinical stratification may be more informative when based on measured functional phenotype rather than diabetes duration alone.

Several important limitations remain. First, human urothelial studies are far fewer than animal studies. Available bladder biopsies are derived largely from patients with diabetes and OAB, and some barrier and sensory abnormalities are similar to those observed in non-diabetic OAB, leaving their diabetes specificity uncertain [54]. Second, a substantial proportion of oxidative-stress, apoptosis, autophagy, and ECM studies use whole-bladder tissue, which precludes definitive localization of the measured changes to the urothelium. Whole-bladder or detrusor readouts therefore support mechanisms at the bladder or tissue level. Direct localization can identify urothelial involvement, but establishing its causal contribution to bladder dysfunction requires cell-specific functional intervention. NLRP3-associated inflammation and recent ferroptosis studies provide more direct urothelial evidence, but the ferroptosis literature remains concentrated in a limited number of studies and a specific molecular axis [51,64,65,69]. Third, most animal studies use independent groups at different time points rather than longitudinally following the same disease course. The precise order in which urothelial, neural, and detrusor abnormalities emerge is therefore difficult to establish, as is whether an individual molecular change is causal, concomitant, or compensatory.

Future work should prioritize these questions of causality and disease-course localization. In animal models, predefined functional states could be evaluated within the same diabetic model by synchronizing voiding behavior and urodynamic testing with assessment of urothelial barrier integrity, ATP and prostaglandin release [99], afferent conduction, detrusor contractility, and ECM changes. Urothelial cell-specific genetic manipulation and lineage tracing could help distinguish the functions of NLRP3, NRF2, and individual cell-death pathways in the urothelium from those in other bladder compartments. Single-cell transcriptomics and spatial omics could further resolve heterogeneity among umbrella, intermediate, and basal urothelial cells and define their spatial relationships with adjacent nerves, immune cells, and the microvasculature. Clinically, longitudinal studies in the same patients should integrate symptoms, urodynamics, post-void residual volume, diabetic complications, and candidate urinary biomarkers to determine whether disease-course-related urothelial changes observed in animal models are also present in humans.

In summary, current evidence most strongly supports a role for the urothelium in early DBD barrier injury, inflammation, and sensory abnormalities, whereas its direct contribution to the late low-activity phenotype remains uncertain. Urothelial barrier, sensory, and paracrine functions do not always change synchronously and do not consistently parallel the overall urodynamic phenotype. Rather than viewing the urothelium as a single driver that determines the transition from a hyperactive to a low-activity bladder, a more evidence-based interpretation is that the urothelium, sensory nerves, detrusor, and local metabolic-inflammatory environment jointly contribute to DBD and that their relative contributions vary across functional states. Defining the temporal order and causal relationships among these changes will be essential for identifying the tissues and processes that dominate bladder dysfunction at different stages and for selecting rational therapeutic targets.

## Figures and Tables

**Table 1:** Disease-course characteristics of DBD functional phenotypes and major tissue changes in different diabetic animal models.

Animal Model	Diabetes Model	Bladder Phenotype and Time Course	Urothelial Changes	Major Neural and Detrusor Changes	Model Limitations	Representative References
STZ rat	Type 1 diabetes-like model induced by STZ-mediated chemical destruction of pancreatic β cells.	Predominantly compensatory changes early; reduced sensation and contractility may emerge with long-standing diabetes.	Early increase in ATP release; intermediate barrier injury; later superficial repair with persistent abnormalities in some sensory receptors.	Abnormal neurotrophic signaling and innervation; long-term detrusor and ECM remodeling.	STZ dose, sex, and observation time can substantially influence the phenotype.	[6,11,26,40,49]
Akita mouse	Type 1 diabetes; Ins2 mutation.	In female mice, OAB predominates at 15 weeks and a UAB phenotype is present at 30 weeks.	Barrier injury at 15 weeks; near-restoration by 30 weeks; late paracrine abnormalities persist.	NLRP3 contributes to changes in bladder nerve density and Aδ/C fibers; FP receptor-mediated contractile responses are enhanced at 30 weeks.	Evidence is concentrated in female mice; a single Ins2 mutation only partially models human T1DM.	[20,51,62,69]
HFD/STZ rat	Type 2 diabetes-like model with insulin resistance and partial β-cell injury.	Compensated state early, with decompensation emerging after approximately 4 months.	Direct disease-course-specific urothelial evidence is limited.	Carbachol- and ATP-induced contractions are enhanced during compensation; ATP responses remain relatively high during decompensation, whereas the cholinergic contractile advantage diminishes.	HFD duration, STZ dose, and dosing protocol can alter metabolic and bladder phenotypes; disease-course urothelial assessment is limited.	[21]
Goto-Kakizaki rat	Non-obese genetic model of type 2 diabetes.	Mild functional changes early; by 46 weeks, bladder capacity and compliance are increased and voiding pressure is reduced.	Disease-course-specific urothelial evidence is limited.	At 46 weeks, afferent conduction is slower, Aδ fibers are reduced, and cholinergic detrusor contraction is impaired.	Does not fully model obesity/metabolic-syndrome-associated T2DM; concurrent urothelial assessment is limited.	[23]
KK-Ay mouse	Obese genetic model of type 2 diabetes.	Hyperresponsive changes predominate at 12–18 weeks; by 25 weeks, voiding efficiency and bladder activity decline.	Barrier function has not been systematically assessed; some sensory- and transmitter-related molecules show disease-course-related changes, but many measurements are from whole-bladder tissue.	Spontaneous detrusor activity is increased early and declines by 25 weeks together with contractile capacity.	Different ages are commonly studied in independent animal groups, and tissue localization of molecular findings is limited.	[22]
FVB db/db mouse	Obese genetic model of type 2 diabetes; Lepr deficiency causes obesity and insulin resistance.	Marked phenotypic differences at 12, 24, and 52 weeks, without a fixed stage sequence.	Urothelial area is increased, but barrier and sensory functions have not been systematically evaluated.	Detrusor area is increased, with collagen accumulation at some time points; disease-course neural changes remain unclear.	Marked sex and interindividual variability; Lepr deficiency and obesity themselves may influence bladder phenotype.	[25]
OLETF rat	Obese genetic model of type 2 diabetes.	Storage abnormalities are present by 36 weeks; contractile function declines further by 48 weeks.	ATP release and VNUT expression are increased, together with reduced bladder blood flow and increased oxidative stress and inflammation.	KCl- and carbachol-induced detrusor contractions at 48 weeks are lower than at 36 weeks.	Age effects cannot be fully separated from diabetes effects.	[60]

Abbreviations: ATP, adenosine triphosphate; DBD, diabetic bladder dysfunction; ECM, extracellular matrix; HFD, high-fat diet; OAB, overactive bladder; STZ, streptozotocin; T1DM, type 1 diabetes mellitus; T2DM, type 2 diabetes mellitus; UAB, underactive bladder; VNUT, vesicular nucleotide transporter.

**Table 2:** Major molecular mechanisms, evidence base, and limitations of pathological urothelial remodeling in diabetic bladder dysfunction.

Mechanism	Urothelial Changes	Relationship to DBD Progression	Evidence Base	Major Limitations	Representative References
Metabolic and oxidative stress	Altered glucose and lipid metabolism, suppression of the NRF2-associated antioxidant response, and increased oxidative/nitrosative stress.	May contribute to barrier injury, cellular stress, and inflammation.	Metabolomic and proteomic analyses of isolated urothelium, together with whole-bladder and detrusor intervention studies.	Whole-bladder or detrusor readouts limit urothelial-specific attribution; links to DBD functional states and disease-course time points remain limited.	[29,70,71,72,73,74,75,76,77]
Mitochondrial injury	Long-standing diabetes is associated with urothelial mitochondrial and other ultrastructural abnormalities.	May contribute to chronic cellular injury.	Primarily morphological evidence from animal urothelium.	Lack of direct measurements of membrane potential, oxygen consumption, and ATP production.	[63,72,73]
Inflammation and pyroptosis	Enhanced NF-κB-associated inflammation; NLRP3 activation accompanied by cell shedding, barrier injury, and pyroptosis-related changes.	Most closely associated with early barrier injury, inflammation, and sensory-nerve changes; may contribute to storage-phase hyperresponsiveness.	Primary urothelial cells, animal studies with whole-animal NLRP3 deletion, and pharmacological intervention.	Whole-animal deletion does not isolate urothelial causality; NF-κB–NLRP3 relationships and differences across functional states remain incompletely defined.	[51,64,69,78,80,82,83]
Ferroptosis	Reduced NRF2/GPX4, iron accumulation, and increased lipid peroxidation; inhibition of ferroptosis attenuates cell injury.	May contribute to urothelial cell injury and inflammation and is linked to high-glucose-associated oxidative damage.	Human DBD tissue, diabetic animals, high-glucose-treated epithelial cells, and intervention/rescue experiments.	Lack of comparisons across DBD functional states and synchronized evaluation with urodynamic phenotypes.	[65]
Apoptosis	Diabetic bladder tissue shows Bax/caspase-, AIF-, and BNIP3-related death signals.	May contribute to chronic diabetic bladder tissue injury.	Evidence derives mainly from whole-bladder tissue, with limited urothelial morphological and cellular-stress data.	Most molecular findings lack urothelial cell-specific localization.	[11,66]
Autophagy and mitophagy	Autophagosomes can be observed in the urothelium during long-standing diabetes, but their functional significance is unclear.	May participate in mitochondrial quality control and cellular injury.	Current DBD urothelial evidence is predominantly morphological.	Direct assessment of autophagic flux and mitophagy is lacking.	[63,73]
Epigenetic and post-transcriptional regulation	H3K9 lactylation and FTO/m6A regulation can suppress NRF2/GPX4; selected circulating miRNAs are associated with bladder fibrosis.	May influence urothelial injury and tissue remodeling by regulating antioxidant capacity and cell fate.	The H3K9la–FTO–m6A–NRF2 axis has animal, cellular, and human-tissue evidence; miRNA evidence derives mainly from circulating exosomes and fibroblasts.	Direct evidence is currently concentrated in a single regulatory axis, and other non-coding RNAs lack urothelial-specific validation.	[65,98]

Abbreviations: AIF, apoptosis-inducing factor; DBD, diabetic bladder dysfunction; ECM, extracellular matrix; FTO, fat mass and obesity-associated protein; GPX4, glutathione peroxidase 4; H3K9la, histone H3 lysine 9 lactylation; m6A, N6-methyladenosine; miRNA, microRNA; NF-κB, nuclear factor kappa B; NLRP3, NOD-like receptor family pyrin domain-containing 3; NRF2, nuclear factor erythroid 2-related factor 2.

## Data Availability

No new data were created or analyzed in this study. Data sharing is not applicable to this article.

## Abbreviations

AGE	advanced glycation end product
AIF	apoptosis-inducing factor
ATP	adenosine triphosphate
DBD	Diabetic bladder dysfunction
DO	detrusor overactivity
DU	detrusor underactivity
ECM	extracellular matrix
EMT	epithelial–mesenchymal transition
FTO	fat mass and obesity-associated protein
GPX4	glutathione peroxidase 4
HFD	high-fat diet
H3K9la	histone H3 lysine 9 lactylation
IDF	International Diabetes Federation
IGF2BP2	insulin-like growth factor 2 mRNA-binding protein 2
MGO	methylglyoxal
m6A	N6-methyladenosine
NF-kB	nuclear factor kappa B
NLRP3	NOD-like receptor family pyrin domain-containing 3
NO	nitric oxide
NOX	NADPH oxidase
NRF2	nuclear factor erythroid 2-related factor 2
OAB	overactive bladder
PGE2	prostaglandin E2
ROS	reactive oxygen species
STZ	streptozotocin
T1DM	type 1 diabetes mellitus
T2DM	type 2 diabetes mellitus
TRP	transient receptor potential
TRPV1	transient receptor potential vanilloid 1
TXNIP	thioredoxin-interacting protein
UAB	underactive bladder
VNUT	vesicular nucleotide transporter
